# Real-world outcomes of COVID-19 treatment with remdesivir in a Spanish hospital

**DOI:** 10.1097/MD.0000000000027228

**Published:** 2021-09-17

**Authors:** Carmen Hidalgo-Tenorio, Coral García-Vallecillos, Sergio Sequera-Arquelladas

**Affiliations:** Unit of Infectious Diseases, Virgen de las Nieves University Hospital/Instituto de Investigación Biosanitario (IBS)-Granada, Spain.

**Keywords:** COVID-19, real-world data, Remdesivir, SARS-CoV-2

## Abstract

Remdesivir is the only antiviral approved for lower respiratory tract infection produced by SARS-CoV-2. The main objective of this study was to determine the mortality rate, readmissions, mean hospital stay, need for higher levels of oxygen support, and adverse effect-induced abandonment rate in hospitalized patients diagnosed with COVID-19 and treated with remdesivir (RDSV). The secondary objective was to determine mortality-related risk factors in these patients.

The study included a prospective cohort of patients admitted to a third level Spanish hospital between July 5, 2020 and February 3, 2021 for COVID-19 diagnosed by SARS-CoV-2 polymerase chain reaction and/or antigen test and treated with RDSV.

Remdesivir was received by 185 patients (69.7% males) with a mean age of 62.5 years, median Charlson index of 3 (interquartile range [IQR]: 1–4), and median ambient air oxygen saturation of 91% (IQR: 90–93); 61.6% of patients had hyper-inflammatory syndrome at admission. Median time with symptoms before RDSV treatment was 5 days (IQR: 3–6) and the median hospital stay was 10 days (IQR: 7–15); 19 patients (10.3%) died after a median stay of 13.5 days (IQR: 9.7–24 days), 58 patients (12.9%) were admitted to ICU, 58 (31.4%) needed higher levels of oxygen support, 0.5% abandoned the treatment due to adverse effects, and there were no readmissions. The only mortality-related factor was the need for higher levels of oxygen support (odds ratio 12.02; 95% confidence interval 2.25–64.2).

All studied patients were admitted to hospital with a diagnosis of COVID-19 and in respiratory failure, needing initial low-flow oxygen support, and all received RDSV within 1 week of symptom onset. The percent mortality was lower in these patients than was observed in all patients with severe COVID-19 admitted to our center (10.3% vs 20.3%, respectively). Despite receiving RDSV, 1 in 3 patients needed higher levels of oxygen support, the sole mortality-related factor.

## 1 Introduction

On December 31, 2019, the World Health Organization (WHO) reported the first case of pneumonia produced by a beta coronavirus similar to Severe Acute Respiratory Syndrome Coronavirus (SARS-CoV) and Middle East Respiratory Syndrome (MERS-CoV), designated as SARS-CoV-2. After the global spread of this virus, initially detected in Wuhan (China), it was named “coronavirus disease 2019” (COVID-19) by the WHO on February 11, 2020.^[1]^ The first case in Spain was reported on January 31, 2020 in the Mediterranean island of Gomera and the second on March 8 in the province of Granada (Southern Spain). In April 2021, it was estimated that the virus had been responsible for 136 million infections and almost 3 million deaths worldwide.^[2]^ Spain has the 9th highest number of notified infections, with an infection prevalence of 2.5%, and around 2 million individuals have been infected to date and >75,000 have died.^[2]^ More than 76,000 infections and 1632 deaths due to COVID-19 have been recorded in the province of Granada.^[3]^

The only antiviral so far approved to treat COVID-19 is the prodrug remdesivir (RDSV), an adenosine nucleoside analog (GS-5734) with broad activity against a wide range of RNA viruses, including those of the *Paramyxoviridae, Coronaviridae, and Filoviridae* families. It directly inhibits viral transcription and replication by acting on RNA polymerase.^[4,5]^ The ACTT-1 trial run by the National Institute of Allergies and Infectious Diseases of North America found that treatment with RDSV versus placebo significantly shortened hospital stay, improved clinical status, diminished the need for higher levels of oxygen (O_2_) support, and reduced the mortality rate in patients requiring low-flow O_2_.^[6]^

The objectives of this study were: to determine outcomes in RDSV-treated patients with severe COVID-19, including hospital stay, ICU admission, need for further higher levels of O_2_ support, mortality, and readmission and to determine mortality-related factors in these patients.

## 2 Patients and methods

### 2.1 Study design and setting

This retrospective study included consecutive patients admitted for COVID-19 and treated with RDSV in a third-level Spanish hospital between July 5, 2020 and February 3, 2021. The pharmacy computer program of the center (PRISMA ATHOS) was used to enroll the patients.

Inclusion criteria were: age >18 years; hospital admission for polymerase chain reaction (PCR)-diagnosed SARS-CoV-2 after ≤7 days with symptoms; meeting at least 2 of the following criteria: respiratory rate ≥24 bpm, O_2_ saturation <94% in ambient air, and/or ratio of arterial O_2_ partial pressure to inspired O_2_ (PaO2/FiO2) <300 mmHg; receipt of at least 1 dose of RDSV during the study period after meeting the following Spanish Health Ministry criteria for its administration, that is, need for low-flow O_2_ therapy (via nasal cannula or simple face mask, with or without reservoir).^[7]^

Exclusion criteria were: severe disease requiring noninvasive ventilation or high-flow O_2_ therapy, invasive mechanical ventilation, or extracorporeal membrane O_2_, the presence of severe liver disease (alanine aminotransfresase or aspartate amonotransferase values ≥5-fold the upper limit of normality or severe renal failure (glomerular filtration <30 mL/min); treatment with hemodialysis or peritoneal dialysis; the need for 2 inotropic agents to maintain arterial blood pressure; pregnancy; breastfeeding status; positive pregnancy test; and evidence of multiple organ failure.

The study was approved by the ethics committee of the hospital, which waived the need for informed consent due to its retrospective and observational design. All information was treated in accordance with Spanish data protection legislation (Law 3/2018, December 5).

### 2.2 Treatment

RDSV was administered following a local protocol based on recommendations from scientific societies and the literature: day 1, bolus dose of 200 mg/iv; days 2 to 5, dose of 100 mg/iv/24 h. The treatment could be prolonged beyond 5 days if deemed appropriate by the attending clinician.

### 2.3 Study variables

Patient epidemiological, clinical, and analytical data were gathered from the DIRAYA computer program of the Public Health System of Andalusia. Information was gathered on age, sex, comorbidities, age-adjusted Charlson comorbidity index, days of hospital stay, days with symptoms before admission, symptomatology, ambient air O_2_ saturation, respiratory rate, SARS-CoV-2 diagnosis by PCR or antigen test, C-reactive protein (CRP) level (as qualitative variable [elevated vs normal] and quantitative variable [mg/dL]); lymphocytopenia (qualitative and quantitative [cells/mL]), thrombocytopenia (qualitative), anemia (qualitative), ferritin (qualitative [< vs ≥ 500 mg/dL] and quantitative [mg/dL]), and d-dimer (qualitative [< vs ≥ 0.5 ng/mL] and quantitative [ng/mL]). The following RDSV-related variables were also collected: days of administration, bolus, and total dose (in milligrams); treatment abandonment and reasons; days between symptom onset and RDSV administration; COVID-19 diagnosis by PCR and/or antigen test; need for higher levels of O_2_ support, need for ICU admission (whether or not actually admitted); mortality; and readmission within 30 days post-discharge.

Information was also collected from the daily report issued by the Department of Preventive Medicine of the hospital on all patients admitted with confirmed or suspected COVID-19 between July 5, 2020 and February 3, 2021, the hospital stay, mortality, discharge, and readmission by care area (normal ward or ICU) (https://www.huvn.es).

### 2.4 Definition of variables

COVID-19: coronavirus infectious disease-2019 pneumonia produced by coronavirus SARS-CoV-2 with pulmonary infiltrates and microbiological confirmation by PCR and/or antigen test.

Hyperinflammatory syndrome or cytokine storm: the presence of pneumonia with SpO_2_ <93% accompanied by ≥2 of the following criteria: temperature >38°C, respiratory rate >24 bpm, PO_2_/FiO_2_ <300; and at least one of the following criteria: IL-6 >40 ng/L, d-dimer >1 mg/L, and ferritin >300 μg/L.^[8]^

Need for higher levels of O_2_ support: requirement for patient to move from support with low-flow O_2_ to high-flow nasal cannula (HFNC), noninvasive/invasive mechanical ventilation, or extracorporeal membrane oxygenation.^[6]^

### 2.5 Sample size

A sample size of at least 151 patients was estimated to obtain an accuracy of 5% in the estimation of a proportion by means of a normal 95% CI, assuming that the proportion was 91% (effectiveness reported in the previous study) and assuming possible losses of 20%.

### 2.6 Statistical analysis

First, means and standard deviations were calculated for quantitative variables. Student *t* test was then applied for independent variables when they were normally distributed (Kolmogorov-Smirnov test) and the Mann–Whitney *U* test when they were not. Absolute and relative frequencies were calculated for qualitative variables followed by application of Pearson *χ*^2^ test or Fisher *χ*^2^ test, as appropriate. Next, multivariate logistic regression analyses were conducted using Freeman formula [n = 10 × (k+1)],^[9]^ entering variables that were significant in bivariate analyses by means of a stepwise approach, with significance levels of 0.05 for entry and 0.10 for exit. The Hosmer-Lemeshow test was used to analyze the goodness of fit of the models. A significance level of 0.05 was considered for all tests. SPSS 21.0 was used for the statistical analysis (SPSS Inc, Chicago, IL).

## 3 Results

### 3.1 Study population

Between July 5, 2020 and February 3, 2021, two thousand six hundred nineteen patients were admitted to our hospital for microbiologically confirmed COVID-19. The present sample comprised 185 (7.1%) of these patients who were treated with RDSV, with a mean age of 62.5 years: 69.7% were male, 84.9% had comorbidities, and their median Charlson index score was 3 (interquartile range [IQR]: 1–4). Table 1 displays results for the remaining variables. As shown in Table 2, the median number of days with symptoms before admission was 5 (IQR: 4–5) and the most frequent symptom was fever (81.6%), followed by cough (72.4%) and dyspnea (69.7%). SARS-CoV-2 diagnosis was by PCR alone in 84.3% and by antigen test subsequently confirmed by PCR in 28.6%. CRP was elevated in 96.2%, ferritin was >500 mg/dL in 60%, D-dimer was >1 ng/mL in 58.4%, lymphopenia was present in 55.7%, and hyperinflammatory syndrome in 61.6% (Table 2).

**Table 1:** Baseline characteristics of the cohort.

Baseline characteristics	N = 185
Age, y, median (±SD)	62.5 (13.16)
Sex	
Female, n (%)	56 (30.3)
Male, n (%)	129 (69.7)
Comorbidities, n (%)	157 (84.9)
Charlson index, median (p25–p75)	3 (1–4)
Arterial hypertension, n (%)	98 (53)
Diabetes Mellitus, n (%)	55 (29.7)
Body mass index >25, n (%)	34 (18.4)
Heart failure, n (%)	14 (7.6)
COPD, n (%)	23 (12.4)
Renal insufficiency grade 3–4, n (%)	3 (1.6)
Solid organ neoplasia, n (%)	16 (8.6)
Hematological neoplasia, n (%)	0
Hematological transplant	0
Solid organ transplant	1 (0.5)

COPD = chronic obstruction pulmonary disease.

**Table 2:** Clinical manifestations and analytical results.

	N = 185
Days with symptoms, median (p25–p75)	4 (3–6)
Symptoms	
Fever, n (%)	151 (81.6)
Dyspnea, n (%)	129 (69.7)
Asthenia, n (%)	114 (61.6)
Cough, n (%)	134 (72.4)
Myalgia, general discomfort, n (%)	77 (41.6)
Diarrhea, n (%)	40 (21.6)
Anosmia, n (%)	14 (7.6)
Dysgeusia, n (%)	15 (8.1)
Respiratory rate, median (P25–P75)	24 (24–26)
Respiratory rate ≥22 bpm, n (%)	102 (55.1)
SO2 AA, median, (P25–P75)	91 (90–93)
SO2 < or = 93%, n (%)	131 (70.8)
COVID-19 diagnosis	
SARS-CoV-2 PCR, n (%)	156 (84.3)
SARS-CoV-2 Ag and PCR, n (%)	53 (28.6)
Analytical disorders	
Elevated C-reactive protein, n (%)	178 (96.2)
C-Reactive protein, mg/dL, mean (±SD)	106.9 (94.6)
Ferritin >500, n (%)	111 (60)
Ferritin, mg/dL, mean (±SD)	1033.9 (1044.9)
d-dimer>1 mg/L, n (%)	108 (58.4)
d-dimer, mg/L, mean (±SD)	1.2 (3.06)
Lymphopenia, n (%)	103 (55.7)
Anemia, n (%)	85 (45.9)
Thrombopenia, n (%)	15 (8.1)
IL-6 >40, n (%)	23/49 (46.9)
Hyperinflammatory syndrome, n (%)	114 (61.6)

IL-6 = interleukinn 6, SD = standard deviation.

Among the 3172 patients admitted to our center with COVID-19 between March 8, 2020 and February 3, 2021, the mean stay in normal ward was 10.6 days, with a crude mortality rate of 20.3%, and the mean stay in ICU was 18.3 days, with a mortality rate of 32.5%. Of these patients, 369 (10.2%) were admitted to the ICU, mainly to receive invasive or noninvasive mechanical ventilation or HFNC.

### 3.2 Remdesivir treatment outcomes

A median of 1 day (IQR: 1–2) elapsed between COVID-19 diagnosis and RDSV administration and a median of 5 days (4–7 days) between symptom onset and RDSV receipt. Nineteen (10.3%) patients died a median of 13.5 days (IQR: 9.7–24) after hospital admission, with 2 in septic shock. The attending physician requested ICU referral for 40 (21.6%) patients but only 24 (12.9%) were actually admitted, with admission being refused for 16 patients (8.6%) due to their present or previous clinical status. Higher levels of O_2_ support were required by 58 patients (31.4%). RDSV was suspended in 1 patient (0.5%) due to transaminase level 5-fold above normal value. No patient (0%) was readmitted (Table 3).

**Table 3:** Remdesivir treatment and outcomes.

	N = 185
Days between RDSV administration and positive diagnostic test, median (p25-p75)	1 (1–2)
Days between RDSV administration and symptom onset, median (p25–p75)	5 (4–7)
Days of RDSV administration, median (IQR)	5 (5–5)
Total dose of RDSV administered, mg (±SD)	524.3 (109.9)
Five-day course of RDSV not completed, n (%)	15 (8.1%)
Withdrawn after admissionto ICU, n (%)	8 (4.3%)
Death, n (%)	2 (1.08)
Improvement in <5 days, n (%)	3 (1.6)
ALT >5-fold above normal value, n (%)	1 (0.5)
Withdrawn by attendingphysician due to respiratory overinfection, n (%)	1 (0.5)
Days of hospital stay, median (p25–p75)	10 (7–15)
Death, n (%)	19 (10.3)
COVID-19 with septic shock, n (%)	2 (1.1)
Days between first RDSV dose and death, median (p25–p75)	12.5 (7–23.5)
Days of hospital stay before death, median (p25–p75)	13.5 (9.7–24)
Need for ICU admission despite RDSV, n (%)	40 (21.6)
Admitted, n (%)	24 (12.9)
Requested admission not accepted due to clinical status of patient, n (%)	16 (8.6)
Need for higher levels of oxygen support after receiving RDSV, n (%)	58 (31.4)
Adverse effects causing RDSV withdrawal, n (%)	
AST elevation	1 (0.5)
Readmissions, n (%)	0

ALT = alanine aminotransfresase, AST = aspartate aminotransferase, ICU = intensive care unit, IQR = interquartile range, RDSV = Remdesivir.

### 3.3 Mortality risk factors in the RDSV Cohort

In the bivariate analysis of results for the RDSV cohort (Table 4), mortality was significantly related to: older age (68.2 vs 61.9 years, *P* = .049); higher median Charlson index score (4 [IQR: 3.5–5] vs 2.5 [IQR: 1–4], *P* = .002); longer mean hospital stay (15 vs 10 days, *P* = .028); the presence of lymphopenia (89.5 vs 51.8%; *P* = .002), thrombopenia (21.1 vs 6.6%), or hyperinflammatory syndrome (89.5 vs 58.4%, *P* = .008); elevated CRP (154.3 vs 101 mg/dL); need for ICU admission (63 vs 16.9%, *P* = .0001); actual ICU admission (42.1 vs 7.8%, *P* = .0001); and need for higher levels of O_2_ support (84.2 vs 25.3%, *P* = .0001) (Table 4). In the multivariate analysis, the only variable significantly related to mortality was the need for higher levels of O_2_ support, that is, invasive or noninvasive mechanical ventilation or HFNC (odds ratio [OR] 12.02; 95% confidence interval [CI] 2.25–64.2) (Table 4).

**Table 4:** Results of bivariate and multivariate analyses of mortality risk factors.

	Survival, N = 166	Death, N = 19	*P* ^∗^	OR (95% CI)
Age, mean (±SD)	61.9 (12.9)	68.2 (14.6)	.049	1.02 (0.95–1.09)
Sex				
Female, n (%)	50 (30.1)	6 (31.6)	.89	
Male, n (%)	116 (69.9)	13 (68.4)		
Comorbidities, n (%)	138 (83.1)	19 (100)	.08	
Charlson index	2.5 (1–4)	4 (3.5–5)	.002	1.57 (0.96–2.57)
BMI >25, n (%)	29 (17.5)	5 (26.3)	.35	
Diabetes mellitus, n (%)	48 (28.9)	7 (36.8)	.47	
Arterial hypertension, n (%)	88 (53)	10 (52.6)	.98	
Heart failure, n (%)	11 (6.6)	3 (15.8)	.16	
COPD, n (%)	20 (12.1)	3 (15.8)	.71	
Renal insufficiency grade 3–4, n (%)	3 (1.8)	0	1	
Solid neoplasia, n (%)	13 (7.8)	3 (15.8)	.22	
Solid organ transplantation, n (%)	1 (0.6)	0	1	
Days of hospital stay, median (P25–P75)	10 (7–15)	15 (9–24)	.028	0.97 (0.91–1.04)
Need for ICU admission, n (%)	28 (16.9%)	12 (63%)	.0001	3.81 (0.71–20.39)
ICU admission, n (%)	13 (7.8)	8 (42.1)	.0001	1.66 (0.22–12.64)
Lymphopenia, n (%)	86 (51.8)	17 (89.5)	.002	11.9 (0.79–181.3)
Lymphopenia, cells/mL, mean (±SD)	1033 (488)	806 (548)	.062	2.5 (0.32–18.4)
Anemia, n (%)	73 (44)	12 (63.2)	.11	
Thrombopenia, n (%)	11 (6.6)	4 (21.1)	.052	0.5 (0.11–2.38)
Elevated CRP, n (%)	159 (95.8)	19 (100)	1	
CRP, mg/dL, mean (±S),	101.5 (90.2)	154.3 (119.1)	.023	0.99 (0.99–1.005)
d-dimer >1, n (%)	97 (59.9)	11 (57.9)	.9	
Ferritin >500, n (%)	98 (59)	13 (68.4)	.43	
Hyperinflammatory syndrome, n (%)	97 (58.4)	17 (89.5)	.008	1.35 (0.192–9.5)
Need for higher levels of O_2_ support, n (%)	42 (25.3)	16 (84.2)	.0001	12.02 (2.25–64.2)
Days with symptoms before RDSV, median (p25–p75)	5 (4–7)	5 (5–6.5)	.103	
Days between diagnosis and RDSV administration, median (p25–p75)	1 (1–2)	2 (1–5)	.62	
Days of RDSV administration	5 (5–5)	5 (5–5)	.62	
Bolus dose not administered (n = 170), n (%)	73 (47.1)	9 (60)	.34	
Total milligrams of RDSV, mean (±SD)	529.5 (105.7)	478.9 (135.7)	.057	1 (0.99–1.005)

BMI = body mass index, CI = confidence interval, CRP = C-reactive protein, OR = odds ratio, RDSV = Remdesivir.

∗ *P* < .005.

## 4 Discussion

This study in a Spanish hospital contributes evidence supporting the clinical benefit of the early administration of RDSV in patients admitted to hospital with COVID19 who require low-flow O_2_ support.

The COVID-19 patients treated with RDSV in our hospital were typically male sexagenarians with comorbidities, most frequently hypertension, diabetes, and overweight. A meta-analysis of 22 studies in patients with COVID-19 found that 40.8% of the patients with COVID-19 had comorbidities and that the mortality rate was 74.3% in this subpopulation; the most prevalent comorbidity was hypertension, followed by diabetes.^[10]^ These conditions are known to upregulate expression of the ACE-2 receptor, increasing the release of proprotein convertase and thereby favoring entry of the virus into host cells.^[11]^

All of the present series of patients had experienced symptoms for less than one week, most frequently fever and cough, and more than three-fifths met the diagnostic criteria for hyperinflammatory syndrome. Almost all patients had elevated CRP levels, and more than half had ferritin levels above 500 mg/dL, d-dimer levels >1 ng/mL, and lymphocytopenia. The hyperinflammatory syndrome is attributable to a cytokine storm, most frequently observed within 1 week of symptom onset. It is associated with increased mortality in these patients through the excess production of proinflammatory cytokines. This leads to the aggravation of adult respiratory distress syndrome, which is present in around 15% of COVID-19 patients and responsible for generalized tissue damage, multiple organ failure, and death.^[12]^ Poor prognostic factors identified in a study of 1449 hospitalized patients with COVID-19 included older age and elevated CRP, d-dimer, and lactate dehydrogenase levels, among others.^[13]^

10% of the RDSV-treated patients in the present investigation did not complete the minimum five days of treatment recommended. Nevertheless, the mean hospital stay of treated patients was only 10 days, 1 patient alone abandoned the treatment, no patient was readmitted, and the mortality was around 10%, much lower than the mortality rate for all COVID-19 patients admitted to our center during the study period. The mortality of RDSV-treated patients was significantly related to the need for higher levels of O_2_ support. These findings are in line with the results of the double-blind, randomized, multicenter ACTT-1 trial, which compared 541 RDSV-treated patients with 521 placebo-treated patients. RDSV treatment was found to shorten the time to recovery by 5 to 7 days (according to the disease severity), reduce the need for mechanical ventilation by 43%; increase clinical improvement by 50%, and reduce mortality by 70% in patients needing only low-flow O_2_.^[6]^ In contrast, the WHO-promoted Solidarity clinical trial reported that RDSV treatment did not reduce the mortality, need for mechanical ventilation, or hospital stay.^[14]^ This discrepancy may be explained by the open and non–placebo-controlled design of the Solidarity trial and by the fact that they only compared between ventilated and nonventilated patients. The nonventilated group included not only patients who did not receive O_2_ support but also those requiring low-flow and high-flow support, associated with different degrees of severity and distinct prognoses. Importantly, the time interval between symptom onset and RDSV administration was not reported, and the drug is known to lose effectiveness from day 11 or 12. Finally, the hospital stay was only calculated indirectly and may have been overestimated, given that recovered patients had to remain hospitalized for 10 days to complete the RDSV schedule.

The present findings are in line with the results of another randomized open trial in hospitalized patients with moderate COVID-19 pneumonia (pulmonary infiltrates and SpO_2_ >94%), which observed a superior clinical status on day 11 after symptom onset in those administered with RDSV for 5 days than in those receiving standard care (OR, 1.65; 95% CI, 1.09–2.48; *P* = .02).^[15]^ However, a randomized, double-blind, placebo-controlled, multicenter trial in China found no difference in mortality, recovery, or hospital stay between 158 COVID-19 patients treated with RDSV and 79 who received placebo, attributed by the authors to the late initiation of the RDSV treatment, which started on day 11 after symptom onset (IQR: 9–12).^[16]^ The latter findings are not comparable with the present results, which were obtained in patients with severe COVID-19 who received RDSV within the first week (mean of 5 days). The early administration of RDSV appears essential to reduce viral replication, the progression of respiratory distress, and the risk of death. RDSV also demonstrated effectiveness when administered on a compassionate-use basis to 53 patients admitted to the ICU with severe COVID-19 (57% initially on mechanical ventilation and 8% on extracorporeal membrane oxygenation); during the 18-week follow-up period, 57% of the patients were extubated, 47% were discharged, and 13% died.^[17]^

Study limitations include the retrospective cohort design and the restriction of RDSV treatment to patients meeting Spanish Health Ministry criteria for its use, limiting the extrapolation of results to other types of patients with COVID-19. The strengths of our study include the large sample size, the standardized treatment protocol applied to all patients, and the reliability of the treatment information, being based on computerized systems that avoid the possibility of missing data.

## 5 Conclusion

Remdesivir was administered to our patients within 24 hours of a COVID-19 diagnosis and after a median of 5 days with symptoms. All patients initially required low-flow O_2_, and one-third needed high-flow O_2_ support. One-fifth of the patients required ICU admission and one-tenth of the patients died during the first 2 weeks of hospitalization, much lower than the global mortality rate for patients with COVID-19 admitted to our center. The sole factor related to mortality was the need for higher levels of O_2_ support.

## Acknowledgments

The authors thank all the staff and “COVID-19 Virgen de las Nieves TEAM” from the University Hospital “Virgen de las Nieves” in Granada, for their immense effort and job carried out in this pandemic; as well as patients and families for their patience and resistance, and especially to those who lost their lives due to SARS-COV2.

## Author contributions

**Conceptualization:** Carmen Hidalgo-Tenorio.

**Data curation:** Coral Garcia-Vallecillos, Sergio Sequera-Arquelladas.

**Formal analysis:** Carmen Hidalgo-Tenorio, Sergio Sequera-Arquelladas.

**Investigation:** Carmen Hidalgo-Tenorio, Coral Garcia-Vallecillos, Sergio Sequera-Arquelladas.

**Methodology:** Carmen Hidalgo-Tenorio, Coral Garcia-Vallecillos.

**Project administration:** Carmen Hidalgo-Tenorio.

**Software:** Coral Garcia-Vallecillos, Sergio Sequera-Arquelladas.

**Supervision:** Coral Garcia-Vallecillos, Sergio Sequera-Arquelladas.

**Validation:** Coral Garcia-Vallecillos, Sergio Sequera-Arquelladas.

**Visualization:** Coral Garcia-Vallecillos, Sergio Sequera-Arquelladas.

**Writing – original draft:** Carmen Hidalgo-Tenorio.

**Writing – review & editing:** Coral Garcia-Vallecillos, Sergio Sequera-Arquelladas.
